# ﻿Development of a multi-entry identification key for economically important fruit fly larvae (Diptera, Tephritidae, Dacinae)

**DOI:** 10.3897/zookeys.1197.116887

**Published:** 2024-04-10

**Authors:** Welma Pieterse, Marc De Meyer, Massimiliano Virgillio, Pia Addison

**Affiliations:** 1 Department of Conservation Ecology and Entomology, Stellenbosch University, Stellenbosch, 7600, South Africa Stellenbosch University Stellenbosch South Africa; 2 Biology Department, Royal Museum for Central Africa, Leuvensesteenweg 13, Tervuren, 3080, Belgium Royal Museum for Central Africa Tervuren Belgium

**Keywords:** Identification tool, interactive key, larvae, LucID, mandible

## Abstract

Identification of fruit fly larvae is difficult due to the limited morphological characteristics present. However, this is the stage at which fruit flies are intercepted at ports of entry through horticultural imports. Molecular tools are useful but are time-consuming and expensive compared to morphological identifications. This project aims to use available information from the literature and our own research to build a multi-entry identification key for thirteen tephritid species and species groups that are of economic concern for the European Union. Third-instar larvae were obtained from different regions and hosts. Thirteen species or representatives of species groups were obtained, including *Ceratitis*, *Dacus*, *Bactrocera* and *Zeugodacus* spp. The cephalopharyngeal skeletons were dissected out, cleared in a 10% NaOH solution, dehydrated and mounted in Euparal on glass slides. Images of at least 20 larvae/species were captured using a compound microscope fitted with a camera. Measurements were taken of the mounted mandibles and the number of tubules and their position in the anterior spiracles in relation to the cephalic skeleton were noted. Differences between morphometric parameters were tested via ANOVA and verified using discriminant function analysis. A matrix was compiled including nine characters for which significant inter-specific differentiation was preliminarily detected. The key was converted into a mobile application by LucID.

## ﻿Introduction

The family Tephritidae has more than 4000 species distributed globally (White and Elson-Harris 1992). The larvae of about 35% of the species attack fruit, including horticultural crops of economic importance (White and Elson-Harris 1992). Fruit flies are among the most destructive pests of these crops and are of quarantine importance for export markets (Ekesi et al. 2006, 2016). As the larvae feed inside fruit, this is the life stage detected during inspection for import or export. However, larvae are difficult to identify morphologically (Frías et al. 2008; Dutra et al. 2012). In the absence of an identification tool to identify the larvae rapidly and quickly, the consignments are usually rejected.

The majority of the thirteen species studied here are at risk of being intercepted when entering Europe through imported fruit, namely, *Ceratitisrosa* s.l. Karsch (Natal fruit fly), *Ceratitiscosyra* (Walker) (Mango fruit fly), *Bactroceradorsalis* (Hendel) (Oriental fruit fly), *Zeugodacuscucurbitae* (Coquillett) (Melon fruit fly) and *Bactrocerazonata* (Saunders) (Peach fruit fly). All are considered potential quarantine pests for the European Union (EU) and are listed as such in the Commission Implementing Regulation 2019/2072 and amending Implementing Regulation 2021/2285. In addition, *B.dorsalis* and *B.zonata* have also been included in the list of priority pests in EU regulation 2019/1702. *Zeugodacuscucurbitae*, *Bactroceraminax* (Enderlein) (Chinese citrus fly), *Bactroceratryoni* (Froggatt) (Queensland fruit fly), *B.dorsalis* and *C.rosa* are listed as A1 quarantine pests in the European and Mediterranean Plant Protection Organization (EPPO) countries (EPPO 2022a). *Bactrocerazonata*, *Ceratitiscapitata* (Wiedemann) (Mediterranean fruit fly) and *Dacusciliatus* Loew (Ethiopian fruit fly) are listed on the A2 EPPO list (EPPO 2022b). *Zeugodacustau* Walker (Pumpkin fruit fly) is mainly a pest of Cucurbitaceae and occurs in Asia and Oceania (Jaleel et al. 2018). Guava and Mango are the main hosts for *Bactroceracorrecta* (Bezzi) (Guava fruit fly), which occurs mainly in Asia (Liu et al. 2019).

Being able to make a correct identification of insect species in the shortest possible period of time is essential to comply with international biosecurity measures, since not all species are of quarantine importance in all countries (Boykin et al. 2012). Several keys for the identification of adult specimens are available (White and Elson-Harris 1992; Virgilio et al. 2014). Descriptions of larvae of some species are given by White and Elson-Harris 1992, but other authors give more detailed descriptions of the larvae of some species (Elson‐Harris 1988; Carroll and Wharton 1989; Carroll 1998; Carroll et al. 2004 onwards; Frías et al. 2008; Lasserre et al. 2009; Steck and Ekesi 2015; Balmes and Mouttet 2017; Dutra et al. 2018a, b; Kamayev et al. 2020; Rodriguez et al. 2021). However, this information is not always easy to access, and the keys are difficult to follow unless specialized expertise is available.

The presence or absence of a preapical tooth on the mandible can be used as a distinguishing characteristic in a taxonomic key, but since the characteristics of the mouthparts of tephtritid species are not known for all species, it could be a controversial character to use. White and Elson-Harris (1992) state that the preapical tooth is absent in third-instar larvae of *C.capitata*, while Carroll et al. (2004) indicate that it might be present in only some specimens. The presence of a preapical tooth on the mandibles is also variable according to Steck and Ekesi (2015), while Balmes and Mouttet (2017) use the presence/absence of the preapical tooth as a diagnostic characteristic in their key. Kamayev et al. (2020) argue that the preapical tooth is too variable to use as a taxonomic characteristic.

According to Pieterse et al. (2017), the shape of the mandibles of fruit fly larvae of *B.dorsalis*, *C.capitata*, *C.rosa* s.s. and *C.cosyra* can be used to distinguish between the third-instar larvae of the species studied. Since shape analysis (which was used in the aforementioned article) is an involved process not suitable for use in a routine diagnostic laboratory, a set of measurements of the cephalic skeleton of third-instar tephritid larvae was designed in the current study that can be used in a robust taxonomic multi-entry key.

Lucid® (https://keys.lucidcentral.org/search/) was developed at the University of Queensland (Norton et al. 2000) and is a multimedia identification and training tool. It is a multi-entry key, as opposed to a traditional dichotomous key, making it more user-friendly for scientists who do not have expert knowledge of the taxonomy of Tephritidae larvae. Multi-entry keys allow the user to choose the characteristics they want to use based on the availability of characters on each specimen. The characters used are also illustrated or imaged for ease of reference. Multi-entry keys have been developed using the LucID platform for adult fruit flies, such as the keys to African frugivorous flies by Virgilio et al. (2014) and the key to adult fruit flies of *Bactrocera* and related genera developed by Doorenweerd et al. (2022).

Multi-entry keys have several benefits over molecular identification tools, namely, they are more accessible and cost-effective; they can be used anywhere without specialised equipment, and the answer is obtained quickly. The key developed here is converted into a mobile application by LucID, for both Android and Apple devices, making it freely available. Multi-entry keys can, furthermore, be used on specimens that are too degraded to be used for DNA analysis. Larval mouth hooks are heavily sclerotized and can still be used even when a specimen is degraded. The aim of the key is to provide a practical identification tool for third-instar fruit fly larvae that are commonly intercepted in the EU.

## ﻿Materials and methods

**Figure 1.**
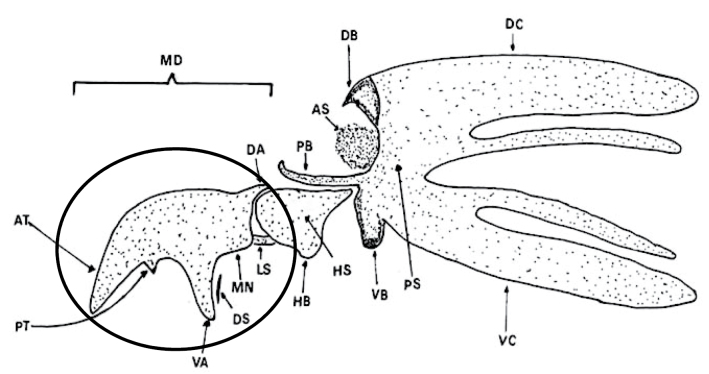
Cephalopharyngeal skeleton of 3^rd^ instar larva, lateral view. Region of interest circled. Abbreviations used in LucID key: AT = Apical Tooth; DA = Dorsal Apodeme; DS = Dental Sclerite; MD = Mandible; MN = Mandibular Neck; PT = Preapical Tooth; VA = Ventral Apodeme (from: Frías et al. 2006).

Preparation of slides. Larvae from 13 species were obtained from colonies from various laboratories around the world and were preserved in 70% ethanol. The heads of the larvae were cut off and cleared by heating in 10% NaOH. The cephalopharyngeal skeletons (Fig. 1) of the larvae of all species were removed, dehydrated with alcohol (70–100%) and mounted in Euparal (Agar Scientific) on glass slides. Images of the slides were captured using a Zeiss compound microscope (MZ 16A) fitted with an Axiocam digital camera (DFC 295). Measurements were taken using ZEN imaging software from the Zeiss MZ 16A.

**Figure 2.**
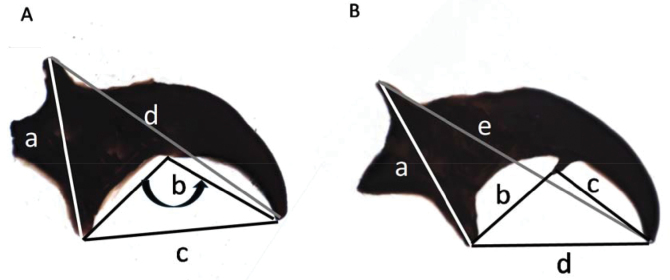
**A, B** Images of a typical tephritid mandible indicating the areas measured. Image (**A**) indicates the measurements for the mandibles without a preapical tooth and (**B**) indicates the measurements for the mandibles where the preapical tooth is present. Measurements are as follows for **A**: (a) the distance between the dorsal apodeme and the ventral apodeme; (b) the ventral angle between the apical tooth and the ventral apodeme; (c) the distance between the ventral apodeme and the apical tooth; (d) the distance between the dorsal apodeme and the apical tooth. Measurements are as follows for **B**: (a) the distance between the dorsal apodeme and the ventral apodeme; b) the distance between the ventral apodeme and the preapical tooth; (c) the distance between the apical tooth and the preapical tooth; (d) the distance between the ventral apodeme and the apical tooth; (e) the distance between the dorsal apodeme and the apical tooth. Measurements were recorded in µm (distances) and degrees (angles).

Fig. 2 shows the various measurements for parameters without (Fig. 2A) and with (Fig. 2B) a preapical tooth.

**Figure 3.**
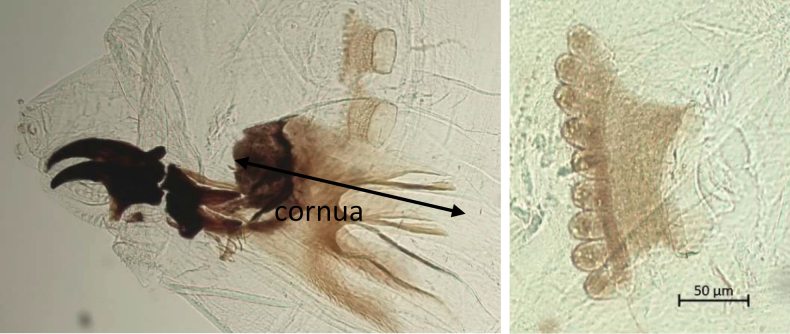
Lateral view of the cephaloskeleton and anterior spiracles of *Bactroceracorrecta* indicating the position of the front spiracles in relation to the cornua (left) and image of the anterior spiracle of *Bactroceradorsalis* showing 9 tubules (right).

Images of the cephaloskeleton and anterior spiracles of ten larvae per species were taken in the same way. The position of the anterior spiracles in relation to the cornua and the number of tubules were recorded for each species (Fig. 3).

### ﻿Data analysis

Discriminant function analysis with classification functions was carried out to statistically allocate the specimens to the species studied using the recorded mandible measurements in Statistica v. 14.1.0 (TIBCO Software Inc, Palo Alto, CA, USA). Measurements for all the specimens of the same species were pooled for these analyses.

## ﻿Results and discussion

A total of 873 mandibles from thirteen species were mounted on slides and examined (Table 1). The raw data of all measurements are listed in the Suppl.material 1.

**Table 1.** Species, origin, and sample size of mandibles used to develop a multi-entry key of tephritid larvae of economic importance to the European Union.

Species	Origin	Number of mandibles measured
*Bactroceracorrecta*	IAEA Vienna colony	51
*Bactroceradorsalis*	Quarantine Station Stellenbosch colony	18
*Bactroceradorsalis*	Atomic Energy Research Establishment Baipayl, Bangladesh colony	13
*Bactroceradorsalis*	IAEA Vienna colony	24
*Bactroceradorsalis*	CRI Nelspruit South Africa colony	24
*Bactroceradorsalis*	University of Pretoria colony	20
*Bactroceraminax*	Changsa, Hunan, China colony	17
*Bactroceraoleae*	IAEA Vienna, colony	29
*Bactroceraoleae*	Madrid, Spain, olives	18
*Zeugodacustau*	IAEA Vienna, colony	20
*Zeugodacustau*	Atomic Energy Research Establishment Baipayl, Bangladesh, colony	22
*Bactroceratryoni*	Queensland University of Technology, Brisbane, Australia, colony	16
*Bactroceratryoni*	IAEA Vienna, colony	37
*Bactrocerazonata*	The “Israel Cohen” Institute for Biological Control, Yehud-Monosson, Israel, colony	24
*Bactrocerazonata*	Atomic Energy Research Establishment Baipayl, Bangladesh, colony	18
*Bactrocerazonata*	IAEA Vienna, colony	14
*Bactrocerazonata*	CIRAD La Réunion, colony	45
*Ceratitiscapitata*	The “Israel Cohen” Institute for Biological Control, Yehud-Monosson, Israel, colony	19
*Ceratitiscapitata*	Plant Quarantine Station, Stellenbosch, South Africa, colony	21
*Ceratitiscapitata*	CRI Nelspruit South Africa, colony	11
*Ceratitiscapitata*	Citrus, Plant Quarantine Station, Stellenbosch, South Africa	4
*Ceratitiscapitata*	Nectarine, Plant Quarantine Station, Stellenbosch, South Africa	8
*Ceratitiscapitata*	CIRAD La Réunion colony	19
*Ceratitiscapitata*	Greece, Bitter orange	11
*Ceratitiscapitata*	CIRAD La Réunion, wild host	18
*Ceratitiscosyra*	CRI Nelspruit South Africa colony	74
*Ceratitisquilicii*	CRI Nelspruit South Africa colony	63
*Ceratitisrosa*	CRI Nelspruit South Africa colony	67
*Dacusciliatus*	Piketberg, South Africa, Pumpkin	19
*Dacusciliatus*	Eduardo Mondlane University, Maputu, Mozambique, Cucumber	20
*Zeugodacuscucurbitae*	Atomic Energy Research Establishment Baipayl, Bangladesh colony	30
*Zeugodacuscucurbitae*	IAEA Vienna colony	20
*Zeugodacuscucurbitae*	CIRAD La Réunion colony	14

We did not see a prominent preapical tooth in any of the mandibles of *C.capitata* third-instar larvae that we examined, so we used the presence/absence of the preapical tooth as one of the distinguishing characteristics in the key. The characteristics and measurements (Table 2), as well as the geographical distribution, the number of tubules and the position of the anterior spiracles in relation to the cornua, were used to compile a LucID key that can be used to identify the third-instar larvae of the species listed in Table 1.

**Table 2.** Average, minimum, and maximum distances (µm) and angles (°) that were used to compile the LucID key for thirteen species of fruit fly larvae of economic importance to the European Union.

Prominent preapical tooth absent				
	***Bactroceradorsalis***	***Bactrocerazonata***	***Ceratitiscapitata***	***Bactroceraoleae***
Distance a (µm)	168 (144–195)	156 (131–171)	141 (130–154)	133 (113–151)
Angle b (°)	102 (87–117)	107 (96–129)	103 (94–116)	103 (93–120)
Distance c (µm)	174 (146–214)	152 (128–180)	130 (113–151)	115 (96–132)
Distance d (µm)	279 (241–326)	252 (203–329)	213 (186–236)	194 (152–215)
	***Bactroceratryoni***	***Bactroceracorrecta***	***Bactroceraminax***	
Distance a (µm)	156 (139–169)	148 (132–161)	278 (253–301)	
Angle b (°)	107 (96–116)	106 (77–120)	110 (101–121)	
Distance c (µm)	156 (144–172)	157 (134–188)	282 (265–303)	
Distance d (µm)	242 (222–262)	257 (232–285)	424 (398–456)	
	***Ceratitisrosa***	***Ceratitisquilicii***	***Ceratitiscosyra***	***Zeugodacuscucurbitae***
Distance a (µm)	149 (134–167)	161 (147–179)	151 (138–168)	194 (163–223)
Distance b (µm)	107 (96–119)	98 (86–110)	96 (82–106)	135 (105–169)
Distance c (µm)	73 (65–84)	74 (64– 79)	79 (69–90)	85 (64–102)
Distance d (µm)	149 (132–171)	148 (127–160)	149 (132–146)	186 (150–225)
Distance e (µm)	233 (202–257)	241 (221–265)	239 (212–263)	299 (245–340)
	***Dacusciliatus***	***Zeugodacustau***		
Distance a (µm)	169 (153–188)	198 (169–224)		
Distance b (µm)	109 (93–121)	139 (112–160)		
Distance c (µm)	47 (40–60)	92 (83–101)		
Distance d (µm)	148 (129–164)	190 (167–211)		
Distance e (µm)	250 (230–267)	315 (279–357)		

*Bactrocerazonata*, *B.tryoni* and *B.correcta* could not be identified reliably without including distribution data as well as the position of the spiracle, indicating a percentage correct identification of below 61% based on the discriminant function analysis (Table 3). *Bactroceracorrecta* was misidentified as *B.zonata* in 27% (14 out of 54) of cases, while *B.zonata* was only identified correctly in 56.43% of cases, often being confused with *B.correcta* and *B.tryoni* (Table 3). However, if the position of the spiracle relative to the cornua was included, *B.correcta* could be distinguished from *B.tryoni* and *B.zonata*. *Ceratitisrosa* and *C.quilicii* can be identified with more than 75% certainty based on the distance between the dorsal apodeme and the ventral apodeme as well as the distance between the ventral apodeme and the apical tooth (Table 4). Overall, it was found that species with a secondary tooth were identified with more accuracy than those without.

**Table 3.** Classification matrix of the species where a secondary tooth is absent on the mandibles. Rows: Observed classifications; Columns: Predicted classifications.

Species	Percent	p-value	*Bd*	*Cc*	*Bz*	*Bo*	*Bt*	*Bc*	*Bm*
	Correct								
*Bactroceradorsalis* (*Bd*)	93.12	0.2985	149	0	4	0	0	7	0
*Ceratitiscapitata* (*Cc*)	97.19	0.1996	0	104	1	1	0	1	0
*Bactrocerazonata* (*Bz*)	56.43	0.1884	6	10	57	0	14	14	0
*Bactroceraoleae* (*Bo*)	80.85	0.0877	0	9	0	38	0	0	0
*Bactroceratryoni* (*Bt*)	49.06	0.0989	0	1	25	0	26	1	0
*Bactroceracorrecta* (*Bc*)	60.78	0.0951	5	1	14	0	0	31	0
*Bactroceraminax* (*Bm*)	100	0.0317	0	0	0	0	0	0	17
Total	78.73		160	125	101	39	40	54	17

**Table 4.** Classification matrix of the species where a secondary tooth is present on the mandibles. Rows: Observed classifications Columns: Predicted classifications.

Species	Percent	P-value	*Cr*	*Cq*	*Cc*	*Zc*	*Dc*	*Bt*
	Correct							
*Ceratitisrosa* (*Cr*)	94.03	0.1988	63	3	1	0	0	0
*Ceratitisquilicii* (*Cq*)	79.36	0.1869	6	50	7	0	0	0
*Ceratitiscosyra* (*Cc*)	66.13	0.1840	9	12	41	0	0	0
*Zeugodacuscucurbitae* (*Zc*)	75	0.1899	0	3	0	48	0	13
*Dacusciliatus* (*Dc*)	100	0.1157	0	0	0	0	39	0
*Zeugodacustau* (*Zt*)	76.19	0.1246	0	0	0	10	0	32
Total	81.01		78	68	49	58	39	45

Balmes and Mouttet (2017) combine morphological characteristics including the presence of a preapical tooth, the number of tubules in the anterior spiracle and the number of oral ridges in their key. We found that it was difficult to consistently produce slide-mounted mandibles of high enough quality to see all the characteristics used. In their description of the third-instar larvae of members of the *Ceratitis* FAR complex, Steck and Ekesi (2015) relied on electron microscope images, which are not practical for use in a routine diagnostic laboratory. Frías et al. (2006, 2008) also used electron microscope images to visualise some characteristics. The multi-entry key published by Carroll et al. (2004 onwards) uses a combination of characteristics that can be observed using a dissection microscope, slides prepared for visualising using a compound microscope and slides prepared to visualise using scanning electron microscopy (SEM). Using this key requires access to an SEM and specialist knowledge of larval morphology.

The LucID key was transformed to an app that can be downloaded from Google Play store (for Android) or Apple App store (for Apple phones). https://play.google.com/store/apps/details?id=com.lucidcentral.mobile.lucid.fruit_fly_larvae&hl=en-US&ah=RArn8-TSJV3KC1m-QBBa0Vfcz7s.

This is the first time a multi-entry key for tephritid larvae of economic significance has been developed in app format. While the characters rely mostly on measurements, it does require some knowledge of how to prepare the mouthparts so that measurements of specific distances can be made. However, it will be a valuable tool for enabling non-molecular identifications of fruit fly larval pests in fruit.

## ﻿Additional information

### Author ORCIDs

Welma Pieterse https://orcid.org/0000-0002-1290-8636

Marc De Meyer https://orcid.org/0000-0003-0755-2898

Massimiliano Virgillio https://orcid.org/0000-0002-1323-6886

Pia Addison https://orcid.org/0000-0002-8227-339X

### Data availability

The raw data supporting the conclusions of this article will be made available by the authors, without undue reservation.

## Supplementary materials

Supplementary material 1 Raw data of all measurements Data type xlsx https://binary.pensoft.net/file/1022998This dataset is made available under the Open Database License (http://opendatacommons.org/licenses/odbl/1.0/). The Open Database License (ODbL) is a license agreement intended to allow users to freely share, modify, and use this Dataset while maintaining this same freedom for others, provided that the original source and author(s) are credited.Welma Pieterse, Marc De Meyer, Massimiliano Virgillio, Pia Addison
